# p47phox: A Central Regulator of NADPH Oxidase Function and a Promising Therapeutic Target in Redox-Related Diseases

**DOI:** 10.3390/cells14141043

**Published:** 2025-07-08

**Authors:** Madison E. Gamble, Sruthi Sureshkumar, Maria Janina Carrera Espinoza, Natalie L. Hakim, Claudia M. Espitia, Fangchao Bi, Kevin R. Kelly, Wei Wang, Steffan T. Nawrocki, Jennifer S. Carew

**Affiliations:** 1 Department of Medicine, University of Arizona Cancer Center, Tucson, AZ 85724, USA; 2 Department of Pharmacology and Toxicology, University of Arizona College of Pharmacy, Tucson, AZ 85721, USA; 3 Division of Hematology, University of Southern California Keck School of Medicine, Los Angeles, CA 90033, USA

**Keywords:** p47phox, oxidative stress, redox, cancer, targeted therapy

## Abstract

The NADPH oxidase 2 (NOX2) complex is a critical regulator of immune homeostasis. It is utilized by phagocytic leukocytes including neutrophils, monocytes, and macrophages to generate reactive oxygen species (ROS) that drive microbe clearance and modulate inflammatory responses. Within NOX2, the essential scaffold protein p47phox plays a pivotal role in orchestrating enzyme activation and facilitating the assembly and membrane translocation of cytosolic components of the complex. Tight regulation of p47phox activity is crucial, and its disruption is linked to a number of pathological conditions. Conversely, its hyperactivity contributes to oxidative stress, tissue damage, the progression of cardiovascular diseases, neurodegenerative disorders, inflammatory conditions, metabolic syndromes, and cancer. In this review, we detail the structural and functional roles of p47phox, mechanisms of its regulation, and its multifaceted contributions to disease pathogenesis. We explore the latest advances in p47phox-targeted therapeutic strategies, discuss current challenges in the field, highlight p47phox’s potential as a transformative target in redox biology and propose future directions to unlock its clinical utility.

## 1. Introduction

Reactive oxygen species (ROS) are critical for the maintenance of immune homeostasis and microbial killing within the phagosome. Phagocytic leukocytes (neutrophils, eosinophils, monocytes, and macrophages) utilize pattern recognition receptors (PRRs) and toll-like receptors (TLRs) to detect pathogens. This primes the NADPH oxidase (NOX) enzyme complex, enhancing its capacity to mount a defensive respiratory burst upon subsequent activation by stimuli such as opsonized microbes or bacterial peptides [1,2]. Superoxide anions (O_2_•^−^) generated by NOX activity are rapidly converted into hydrogen peroxide (H_2_O_2_), hydroxyl radicals (OH•), and hypochlorous acid (HOCl) via secondary enzymatic reactions [3,4]. This process is normally tightly controlled, but its dysregulation can lead to oxidative damage to host tissues, chronic inflammation, cellular senescence, and a wide variety of diseases [5,6].

Phagocytes preferentially utilize the NADPH oxidase complex to generate reactive oxygen species (ROS) for host defense. This multicomponent enzyme system—often referred to as NOX2—includes the membrane-bound catalytic core (gp91phox, also known as NOX2, and p22phox), cytosolic regulatory subunits (p47phox, p67phox, p40phox) and regulatory GTPases such as Rac1/2 [7,8,9]. The scaffold protein p47phox (neutrophil cytosolic factor 1, *NCF1)* plays a central role in NOX2 activation through its conformational dynamics. When at rest, it adopts an autoinhibited conformation that prevents unwanted basal ROS production [10]. Invading pathogens and inflammatory signals trigger the serine phosphorylation of p47phox (Ser303–Ser379), which induces a conformational change that exposes its phox homology (PX) and Src homology 3 (SH3) domains. This facilitates membrane translocation and assembly of the NOX2 complex [11].

Given p47phox’s role as a key determinant of NOX2 activity, it is not surprising that its dysfunction has been linked to immunodeficiency and hyperinflammatory states [12]. For example, mutations or deletions in *NCF1* cause a rare genetic disorder called chronic granulomatous disease (CGD) that is associated with defective ROS production, recurrent infections, and granuloma formation [13,14]. On the flip side, excessive p47phox-driven oxidative stress contributes to atherosclerosis, Parkinson’s disease, Alzheimer’s, rheumatoid arthritis (RA), diabetes, cancer, etc. [6,9,15]. p47phox’s important role in so many diseases creates opportunities for innovative precision therapeutic strategies to inhibit or activate its function where appropriate.

This review provides an in-depth analysis of p47phox’s structural architecture, its mechanisms of action and contributions to various disease states. We highlight recent advances in p47phox-targeted therapies, address current challenges in the field and propose future directions for research and clinical translation. Drawing on the latest literature, we position p47phox as a key player in redox biology and an emerging target for precision medicine in oxidative stress-related disorders.

## 2. Structural Features of p47phox

**Figure 1:**
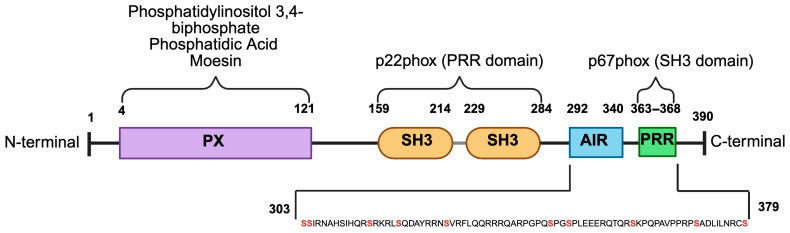
Structure of human p47phox. p47phox contains one phox homology (PX) domain, two SRC homology (SH3) domains, one autoinhibitory region (AIR), and a proline-rich region (PRR). Several serines (marked in red) in the C-terminal region may be phosphorylated to induce activation.

p47phox is a 390-amino-acid protein encoded by the *NCF1* gene located on chromosome 7q11.23. It is highly abundant in neutrophils (100–150 ng per million cells) to ensure rapid NOX2 activation during immune challenges [16]. The structure of p47phox is comprised of multiple functional domains, with each contributing to its regulatory and interactive roles within the NOX2 complex (Figure 1). The N-terminal PX domain (residues 4–121) facilitates membrane association during NOX2 activation through specific binding to phosphoinositides, particularly phosphatidylinositol 3,4-bisphosphate (PtdIns(3,4)P_2_). Unlike the PX domain of p40phox, which binds phosphatidylinositol 3-phosphate (PtdIns(3)P), the PX domain of p47phox shows minimal affinity for PtdIns(3)P, but may also interact with phosphatidylserine (PS) or phosphatidic acid (PA) [17,18,19]. The PX domain is followed by two tandem Src homology 3 (SH3) domains that enable protein–protein interactions that are essential for NOX2 function. The SH3A (residues 159–214) and SH3B (229–284) domains stabilize the membrane-bound NOX2 core by binding to the proline-rich region (PRR) of p22phox [20,21]. Mutations within this region disrupt p47phox–p22phox interactions and severely impair superoxide production. Key examples include the Δ219–222 deletion (reduces superoxide generation by up to 60%) and the W193R substitution (completely inhibits superoxide production) [22,23]. These findings highlight the structural and functional importance of the p47phox SH3 domains as regulators of NADPH oxidase activity. The C-terminal autoinhibitory region (AIR, residues 292–340) maintains p47phox in a closed, inactive conformation by masking its interaction domains to prevent premature NOX2 activation [24]. Finally, the C-terminal proline-rich region (PRR, residues 363–368) interacts with the SH3 domain of p67phox to facilitate the pre-assembly of the cytosolic complex (p47phox-p67phox-p40phox) before membrane recruitment [25,26].

Structural insights into p47phox have primarily been gained through X-ray crystallography and nuclear magnetic resonance (NMR) studies, which have elucidated the conformation of its key domains in both autoinhibited and active states. The PX domain structure was resolved by X-ray crystallography and revealed a conserved phosphoinositide-binding pocket critical for membrane targeting [19]. The tandem SH3 domains have been characterized by crystallography, showing a compact fold that enables simultaneous interaction with the p22phox PRR and regulatory flexibility [24]. Small-angle X-ray scattering (SAXS) and hydrogen-deuterium exchange mass spectrometry (HDX-MS) have further contributed to understanding the conformational dynamics of full-length p47phox during phosphorylation-induced activation [27]. While a high-resolution structure of the full-length protein remains unavailable, integrative modeling and molecular dynamics simulations have provided evidence for domain rearrangements and allosteric regulation, particularly involving the autoinhibitory AIR domain and its release upon serine phosphorylation [28]. These studies collectively underscore the modular and dynamic nature of p47phox, which enables precise spatiotemporal regulation of NOX2 activity in immune responses. Moreover, these efforts identified transient allosteric sites that could potentially be targeted for therapeutic intervention [27,28,29].

## 3. Molecular Mechanisms of p47phox in NOX2 Activation

### 3.1. Phosphorylation and Conformational Change

p47phox adopts a compact, autoinhibited conformation in its resting state [10]. NOX2 activation occurs at phagosomal or plasma membranes where the cytochrome b558 complex (gp91phox and p22phox) is located. Activation is highly regulated and involves the transfer of electrons from NADPH to molecular oxygen to generate superoxide [1,5]. In resting neutrophils, the cytosolic components (p47phox, p67phox, p40phox, and Rac2) remain sequestered in the cytoplasm to maintain NOX2 in an inactive state. This prevents basal ROS production and oxidative damage to host tissues [7]. Priming of phagocytes is initiated by pathogen-associated molecular patterns (PAMPs), damage-associated molecular patterns (DAMPs), or inflammatory cytokines. This prepares the NADPH oxidase complex for rapid activation upon subsequent stimulation by agents such as the bacterial peptide formyl-methionyl-leucyl-phenylalanine (fMLF).

**Figure 2:**
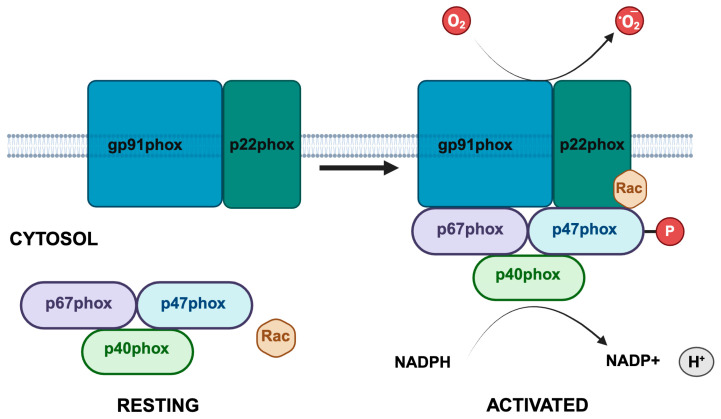
Schematic representation of NOX2. p47phox, p67phox and p40phox remain in the cytosol in a resting state (left side). Upon cell stimulation, p47phox, p67phox and p40phox along with the small G-protein Rac attach to membrane-bound cytochrome b558 (gp91phox and p22phox), and NOX2 becomes activated. Phosphorylation of p47phox is a key step in the activation process that induces the conformational changes required for activation and production of superoxide.

The functional activity of p47phox is tightly regulated by a complex network of phosphorylation events, PTMs and protein–protein interactions (Table 1). This process is essential for redox homeostasis and ensures precise control of NOX2 activation and ROS production. Activation begins with phosphorylation of its C-terminal serine-rich region (Ser303–Ser379), which acts as a molecular switch for NOX2 assembly [30]. This can be mediated by PKC isoforms (α, βII, δ, ζ), ERK1/2, Akt, p38 MAPK, and others [31]. Phosphorylation of key residues such as Ser379 within the conserved “RRXSXR” motif induces a conformational shift that disrupts the AIR to expose the PX and SH3 domains. This enables p47phox to bind PtdIns(3,4)P_2_ at the membrane and interact with p22phox via its SH3 domains [10,20]. The resulting conformational change transforms p47phox from a compact, functionally inactive state into an extended, functionally active form that facilitates the recruitment of p67phox and p40phox to form the active NOX2 complex (Figure 2) [32]. Earlier studies showed that treatment with phorbol 12-myristate 13-acetate (PMA) rapidly leads to Ser379 phosphorylation-driven NOX2 activation [32]. Additional in vitro investigations demonstrated that PKC-mediated phosphorylation of recombinant p47phox in the presence of p67phox and Rac2 was sufficient to trigger an oxidative burst. This study validated its role as a master regulator of the complex [30]. While Ser379 is the most well-characterized functional activation-linked p47phox phosphorylation site, recent phosphoproteomic analyses have identified additional potential sites for PKC-driven functional activation, including Ser345. Initial evidence suggests that phosphorylation of these less intensively studied sites may enhance p47phox membrane affinity under specific inflammatory conditions [33]. Additional studies are required to better define these dynamics.

The current working model of kinase-driven NOX2 regulation involves PKC driving the rapid, acute phase of ROS production with AKT and MAPKs sustaining and amplifying the response over time. This coordinated interplay ensures a balanced and adaptable oxidative response. Evidence suggests that AKT-driven phosphorylation of p47phox on Ser304 and Ser328 stabilizes its active conformation to promote sustained ROS generation [34]. p38 MAPK and ERK1/2 can also modulate NOX2 activity via Ser345 phosphorylation. This enhances p47phox responsiveness to secondary stimuli, such as fMLF. Pro-inflammatory cytokines like TNF-α act as priming agents, amplifying NOX2-dependent ROS production during subsequent microbial encounters. This mechanism is heavily utilized by neutrophils for antimicrobial defense [33].

The kinase-related regulatory landscape of NOX2 activity is further complemented by protein kinase A (PKA) and casein kinase 2 (CK2). PKA has been specifically implicated in phosphorylating p47phox at distinct sites (e.g., Ser320) in response to cyclic AMP signaling [35,36]. CK2 may also modulate p47phox functional activity under specific stress conditions [37]. The complexity of kinase-mediated regulation of p47phox underscores its role as a molecular rheostat in NOX2 activation. Indeed, excessive kinase activity or impaired dephosphorylation have both been linked to chronic inflammatory diseases and oxidative stress-related disorders. The improved understanding of how this kinase network works has highlighted clear opportunities for precision modulation of ROS production.

### 3.2. Role of Cytosolic Regulators

Once phosphorylated, p47phox serves as a scaffold that recruits p67phox and p40phox to membrane-bound cytochrome b558. The C-terminal PRR of p47phox binds the SH3 domain of p67phox. The complex is then stabilized through the binding of p40phox to the PB1 domain of p67phox [18,24]. NOX2 stability and catalytic efficiency are further enhanced when GTP-bound active Rac2 binds to gp91phox and p67phox. This ensures robust electron transfer and superoxide production [34]. Recent studies using fluorescence resonance energy transfer (FRET) have mapped these interactions in real time. Molecular dynamics simulations and AlphaFold-predicted structures have provided further insights into the assembly and activation of the NOX2 complex [28,38]. These findings support a model in which cytosolic subunits (p47phox, p67phox, and p40phox) translocate as a pre-assembled complex, while Rac2 is recruited independently and plays a key role in stabilizing and orienting the NOX2 complex at the membrane.

### 3.3. Efficiency of NOX2 Activation

Although only a fraction of cytosolic p47phox actually translocates to the membrane upon stimulation, this is sufficient to trigger substantial superoxide generation [8]. This efficiency is attributed to the synergistic dynamics between p47phox, p67phox and Rac2 in NOX2 assembly and stabilization. Mutagenesis studies have confirmed the essential role of p47phox in the NOX2 activation process. For example, a serine to alanine mutation at position 379 completely impairs NOX2 activity. Dual Ser303/Ser304 mutations dramatically diminish superoxide production [30]. These collective findings underscore the importance of these specific phosphorylation sites as regulators of the complex. Additionally, computational modeling and inhibitor studies (e.g., C6 and C14) have identified key molecular interfaces, including gp91phox regions (residues 86–93, 450–457), that indirectly influence NOX2 regulation via p22phox. These interfaces could represent potential targets for therapeutic intervention [39,40].

### 3.4. Role of Pin1

The interaction of prolyl isomerase Pin1 with phospho-Ser345 of p47phox induces a conformational change that exposes additional serine residues for PKC phosphorylation to amplify NOX2 activity [19]. This is another example of the regulatory feedback loop of phosphorylation-dependent structural changes that titrate p47phox function. Recent studies suggest that Pin1 promotes p47phox activation by facilitating conformational changes necessary for NOX2 complex assembly. This interaction plays an important regulatory role and may represent a potential therapeutic target for modulating ROS production [41]. Additional layers of regulation are achieved through the binding of 14-3-3 proteins to phosphorylated p47phox. This binding stabilizes its active conformation, while its dephosphorylation by the PP2A phosphatase inhibits NOX2 activity [42].

### 3.5. Other Post-Translational Modifications

In addition to phosphorylation, p47phox is regulated by several other post-translational modifications (PTMs) that influence its stability, interactions, and activity within the NOX2 complex. For example, p47phox levels can be titrated during prolonged immune responses to modulate ROS production through lysine-targeted ubiquitination to degrade a portion via the ubiquitin–proteasome system (UPS) [43]. Conversely, lysine acetylation mediated by enzymes like p300 offers another mechanism to control p47phox stability and promote its association with other NOX2 subunits under oxidative stress conditions [44]. ROS-triggered S-glutathionylation of cysteine residues may protect p47phox from irreversible oxidative damage or fine-tune its activation in response to redox stress [45]. Additionally, S-nitrosylation has been proposed as a mechanism by which nitric oxide signaling could inhibit NOX2 activity through p47phox modification [46]. Finally, O-GlcNAcylation has emerged as a potential regulatory mechanism linking metabolic cues to p47phox function [47]. Collectively, these PTMs underscore the multifaceted regulation of p47phox and present opportunities for therapeutic intervention in redox-driven diseases.

## 4. p47phox in Pathological Conditions

### 4.1. Chronic Granulomatous Disease (CGD)

Chronic granulomatous disease (CGD) is a rare primary immunodeficiency. It affects approximately 1 in 200,000 individuals worldwide and is characterized by impaired ROS production that results from NOX2 defects [13]. *NCF1* mutations account for approximately 25% of all CGD cases [48]. The most common *NCF1* mutation in patients with CGD is a deletion of GTGT in exon 2. This deletion results in a frameshift that prematurely terminates the protein and impairs superoxide generation [49]. Consequently, phagocytes are left unable to kill pathogens such as *Staphylococcus aureus*, *Aspergillus*, *Burkholderia cepacia*, and *Nocardia*. Affected patients experience recurrent, life-threatening infections [50]. The severe immunodeficiency caused by *NCF1* defects translates into most CGD patients being diagnosed at an early age. They typically begin experiencing abscesses, pneumonia, and fungal infections along with chronic inflammation-induced granuloma formation by age 10 [51]. The increased susceptibility of CGD patients to infections is linked to the redox-related downstream consequences of p47phox deficiency. The impaired ability to generate superoxide means that these patients also produce very low levels of hydrogen peroxide and hypochlorous acid, both of which are critical for microbial killing [52]. Beyond their primary neutrophil defects, CGD patients also experience additional immune dysfunction due to impaired macrophage antigen presentation [53]. The complexities of the immunobiology of CGD have been defined largely through studies with *NCF1^−^/^−^* mice, which faithfully replicate the primary features of human CGD. Like humans with CGD, *NCF1^−^/^−^* mice exhibit granuloma formation, increased IL-1β and TNF-α production, and heightened susceptibility to *Aspergillus* infections [54]. A recent study linked p47phox deficiency in CGD to increased microbiota-related susceptibility to colitis. The investigators demonstrated that intestinal epithelial cells from *NCF1^−^/^−^* mice produced increased levels of ROS as compared with gp91phox^−^^/−^ mice. Their findings suggest that the susceptibility of CGD patients to colitis development is driven in a genotype-specific manner [55]. Not only does the ability to model the disease in mice offer an opportunity to mechanistically investigate the complex physiology of CGD, it also provides a platform to preclinically assess novel therapeutics to better manage the disease (Table 2).

Current treatment strategies for CGD focus on improving immune defense through prophylactic antibiotics, antifungals, and interferon-γ, but do not correct the underlying deficiency in ROS production that drives disease pathology [54]. Gene therapy using lentiviral vectors to deliver functional *NCF1* has shown promise, achieving partial restoration of ROS production in patient-derived neutrophils and a reduction in infection rates in preclinical models [56]. Clinical investigation of the safety and efficacy of this approach has only recently been initiated [57].

### 4.2. Cardiovascular Diseases

p47phox-driven oxidative stress has emerged as a central player in cardiovascular diseases including atherosclerosis, hypertension, and endothelial dysfunction [58]. The production of ROS in the vascular wall sets off a cascade of damage. The oxidization of low-density lipoprotein (LDL) cripples nitric oxide (NO) signaling and sparks the inflammation that ultimately leads to plaque buildup [59,60]. The accumulation of LDL particles in arterial walls transforms macrophages into foam cells that pile up lipids and form the fatty streaks of early atherosclerosis [61]. This process ultimately culminates in the hardening and narrowing of arteries.

Studies in mice have painted a striking picture of the true impact of p47phox on cardiovascular pathogenesis. The infusion of angiotensin II into mice lacking p47phox expression results in a significant drop in vascular superoxide levels compared to their wild-type counterparts. Their systolic blood pressure returned to the near normal range and their atherosclerotic plaques also shrank [62,63]. These findings suggest that p47phox acts as an architect of vascular damage in hypertension and atherosclerosis. The increased basal ROS levels in endothelial cells that are triggered by p47phox accelerate plaque formation [64]. This mechanistic relationship offers an opportunity for therapeutic intervention. Antioxidants like Ginsenoside Rb1 can reduce superoxide production and improve vascular function [65]. While not curative, this type of approach could be helpful in controlling disease progression. Additionally, a recent study linking the lysine methyltransferase SET7 to NOX-driven atherosclerosis suggests that epigenetic targeted therapies might also aid in managing the disease [66].

NO is critically important for keeping blood vessels structurally flexible. This prevents monocytes from sticking and triggering inflammation. Unfortunately, p47phox hyperactivity antagonizes the benefit of NO in this context. When p47phox-driven ROS oxidizes NO or its precursors, it reduces NO availability. This leads to stiffened vessels and the recruitment of inflammatory cells [67]. Deletion of p47phox (*NCF1*) in the *ApoE* knockout mouse model of atherosclerosis reduces aortic ROS levels and shrinks plaque size [68]. These findings demonstrate the pathological significance of unchecked p47phox activity on cardiovascular homeostasis. Indeed, a study of patients with advanced atherosclerotic lesions revealed that they often have higher levels of *NCF1* mRNA in their plaques [69]. There is also evidence that *NCF1* gene variants may contribute to hypertension by influencing ROS production in vascular cells. However, their specific impact on stroke risk remains under investigation [70].

The role of p47phox in cardiovascular disease is ultimately one of balance. While it is essential for fighting pathogens, it can be incredibly destructive when functionally hyperactive in blood vessels. As we continue to define its role in cardiovascular pathogenesis, we will be able to harness that knowledge to design precision therapies. Inhibiting it could antagonize the oxidative stress that drives cardiovascular damage, but care needs to be taken to ensure that doing so does not compromise immune function. Modulating upstream pathways like angiotensin II signaling or glucose metabolism that amplify p47phox may offer an alternative approach [71]. There is also growing interest in the potential use of novel antioxidants or compounds that stabilize NO signaling to complement p47phox modulation [72]. Further investigation is required to ultimately determine which strategy is optimal for individual patients. This will likely depend largely on their overall health status as comorbidities could significantly change the landscape.

### 4.3. Neurodegenerative Diseases

p47phox has emerged as a driver of oxidative stress, neuroinflammation and neuronal loss in conditions like Parkinson’s disease (PD) and Alzheimer’s disease (AD) [73]. By orchestrating excessive ROS production in activated microglia, p47phox initiates a cascade that damages the delicate architecture of the brain. p47phox is also linked to the destruction of dopaminergic neurons in the substantia nigra, the region of the brain that is critical for motor control in PD. p47phox-mediated ROS also promote the aggregation of α-synuclein, which is a hallmark feature of PD. This oxidative onslaught also disrupts mitochondrial homeostasis by triggering membrane depolarization, cytochrome c release, and neuronal cell apoptosis [74,75]. Studies using MPTP (1-methyl-4-phenyl-1,2,3,6-tetrahydropyridine), a toxin that mimics PD in mice, demonstrated that mice lacking the *NCF1* gene retain significantly more dopaminergic neurons than their wild-type counterparts. They also exhibit superior motor coordination and lower levels of oxidative damage markers such as 4-hydroxynonenal (4-HNE) [76]. These studies suggest that inhibiting p47phox activity may be sufficient to antagonize PD progression. p47phox also contributes to the pathogenesis of AD through other mechanisms. ROS generated by p47phox-activated microglia trigger the hyperphosphorylation of the tau protein and amyloid beta (Aβ) plaque buildup [77]. These alterations cause synaptic dysfunction and erode cognitive abilities. In the *APP/PS1* mouse model of AD, knocking out p47phox is sufficient to limit tau pathology [78]. These findings suggest that p47phox dysfunction plays a pivotal role in AD pathogenesis [79].

The therapeutic potential of targeting p47phox for the treatment of neurodegenerative disorders is scientifically rational. Small molecule NOX2 inhibitors like Ebselen have shown promise in preclinical models of PD and AD. Their ability to reduce oxidative stress leads to an increase in in vitro neuronal cell survival and reduced inflammation and neuronal loss in mouse models of disease [80]. However, the safety and efficacy of long-term p47phox inhibition remains uncertain. Since NOX2 helps to clear debris and fight infections in normal microglial cells, it is possible that chronic suppression could disrupt brain homeostasis or increase vulnerability to pathogens [81]. In view of this, alternative strategies to counter the pathogenic effects of p47phox are being explored. Modulation of upstream signals that amplify p47phox activity like angiotensin II and inflammatory cytokines are being investigated in neurodegenerative contexts [82]. Others are evaluating the potential benefit of antioxidants tailored to neutralize p47phox-derived ROS without broadly impairing NOX2 [83]. A deeper understanding of the mechanisms by which p47phox interacts with α-synuclein, tau, and Aβ may facilitate a balanced strategy that does not compromise the brain’s defenses.

### 4.4. Inflammatory Disorders

p47phox plays a pivotal yet dichotomous role in the pathogenesis of inflammatory disorders. In rheumatoid arthritis (RA), p47phox drives synovial inflammation and joint destruction. Excessive p47phox-mediated NOX2 activation generates a surge of ROS within the synovium, which causes oxidative damage to cartilage and bone [84,85]. The relationship between p47phox and disease activity has been validated in synovial biopsies from RA patients, which demonstrated elevated p47phox expression and significant superoxide generation [86]. Additional studies have shown that the peptidyl-prolyl isomerase Pin1 enhances p47phox phosphorylation to amplify NOX2 activity in RA [87]. This hyperactive state sustains ROS production while also activating the NF-κB signaling pathway to drive the expression of pro-inflammatory cytokines like interleukin-6 (IL-6) and TNF-α (Table 3) [88]. Preclinical studies in collagen-induced arthritis models established proof of concept for therapeutic Pin1 inhibition. Synovial ROS levels were reduced, cartilage erosion was limited and NF-κB-driven inflammation was suppressed in response to Pin1 inhibition [89]. These findings suggest that targeted inhibition of p47phox or its upstream regulators could offer novel avenues for disease management.

Conversely, p47phox plays a protective role in inflammatory bowel disease (IBD) by maintaining gut homeostasis. In the intestinal mucosa, ROS contribute to microbial killing and the reinforcement of the integrity of the epithelial barrier. Both are critical defenses against dysbiosis and inflammation [90]. Studies in *NCF1*^−^/^−^ mice revealed that p47phox deficiency resulted in increased susceptibility to colitis in a manner that was directly influenced by the microbiome [91]. This provided further evidence that p47phox activity is required for gut immune homeostasis [92]. Reduced *NCF1* expression is also associated with more frequent flares in patients with Crohn’s disease. This is likely due to impaired microbial control. Similarly, elevated *NCF1* expression is correlated with greater disease severity in patients with ulcerative colitis. This has been attributed to excessive ROS-driven inflammation [93]. Collectively, these studies illuminate the paradoxical role of p47phox in gut immunity, where both under- and overactivity can precipitate pathology. Beyond RA and IBD, emerging evidence implicates p47phox in other inflammatory conditions, such as systemic lupus erythematosus (SLE) and psoriasis. In SLE, *NCF*1 polymorphisms are linked to increased disease susceptibility due to p47phox-driven ROS-mediated autoantigen generation and immune complex-mediated tissue damage [94]. Similarly, in psoriasis, p47phox activation in keratinocytes amplifies ROS-dependent inflammatory signaling. This exacerbates epidermal hyperplasia [95]. These examples further illustrate the context-specific nature of p47phox’s contributions to inflammation.

Therapeutically, modulating p47phox activity in inflammatory diseases presents both opportunities and challenges. In RA, inhibitors targeting NOX2 or its upstream activators like Pin1 or PKC may offer an opportunity to reduce synovial oxidative stress without broadly suppressing immunity. Small molecule NOX2 inhibitors have shown efficacy in reducing joint inflammation in preclinical models of the disease [96]. However, their specificity and long-term safety require further validation. In contrast, approaches to enhance p47phox function in IBD like Crohn’s disease could potentially restore microbial homeostasis. To be successful, such strategies would need to avoid triggering excessive ROS production as that could exacerbate inflammation. Precision medicine approaches guided by genetic profiling of *NCF1* variants or tissue-specific ROS profiling could help tailor interventions to individual disease contexts. The current evidence in the field indicates that p47phox acts as a molecular fulcrum that balances protective and pathological outcomes. As continued research further defines the molecular and contextual determinants of p47phox activity, precision therapeutics that fine-tune ROS production in a disease-specific manner may become a reality.

### 4.5. Metabolic Disorders

p47phox-driven ROS play a pivotal role in metabolic diseases such as diabetes mellitus by driving the underlying oxidative stress and tissue damage that contribute to pathogenesis in multiple organ sites. In type 1 and type 2 diabetes, hyperglycemia activates p47phox to amplify ROS production via multiple signaling pathways including PKC, MAPK, and the renin–angiotensin system. This impairs insulin signaling, glucose uptake, and mitochondrial function [97]. The resulting oxidative burden disrupts redox homeostasis and promotes lipid peroxidation, protein carbonylation, and DNA damage in pancreatic β-cells, adipocytes, hepatocytes, and vascular endothelial cells. Ultimately, this exacerbates insulin resistance and triggers β-cell apoptosis [98].

Diabetic retinopathy is a leading cause of blindness in diabetic patients. It has also been linked to p47phox-driven oxidative stress in retinal endothelial cells and pericytes. Hyperglycemia-induced p47phox activation has been shown to double vascular ROS levels and trigger endothelial dysfunction, pericyte loss, and neovascularization [99]. The related retinal pathology has been tied to the induction of vascular endothelial growth factor (VEGF) and matrix metalloproteinases (MMPs), which heighten vascular permeability and angiogenesis [100]. In mouse models of diabetic retinopathy, photoreceptor-driven oxidative stress via NOX contributes to ROS generation, retinal vascular leakage, and capillary degeneration. This suggests a potential role for p47phox in these pathologies and indicates that therapeutic p47phox inhibition may hold promise as a novel approach to preserve vision in diabetic patients [101].

p47phox hyperactivation is also a contributing factor in diabetic renal dysfunction. In glomerular mesangial cells and podocytes, increased ROS levels have been linked to extracellular matrix accumulation, fibrosis, and albuminuria [102]. p47phox-mediated ROS production has been shown to mediate these pathogenic effects through the activation of TGF-β and NF-κB signaling pathways. This promotes the pro-fibrotic and pro-inflammatory responses that underlie glomerulosclerosis [103]. In further support of this deleterious relationship, studies in *NCF1^−^/^−^* diabetic mouse models demonstrated that p47phox deficiency results in a reduction in glomerular ROS, decreased albuminuria, and improved renal function [104]. p47phox-mediated ROS generation in peripheral nerves and Schwann cells is also believed to contribute to diabetic neuropathy by promoting axonal degeneration and sensory loss. Mechanistically, p47phox has been hypothesized to promote neuropathy in this context through ROS-mediated activation of poly(ADP-ribose) polymerase (PARP) and caspase-3, which stimulates neuronal apoptosis and mitochondrial dysfunction [99].

Although the scientific support for a role for p47phox in the pathogenesis of metabolic disorders is currently strongest in diabetes, there is growing evidence that it also contributes to the development or progression of other metabolic diseases. Non-alcoholic fatty liver disease (NAFLD) and obesity are two examples of this where ROS generation in hepatic and adipose tissue drive insulin resistance and steatosis [105,106,107]. Collectively, these findings indicate that tightly controlled p47phox activity is required to maintain metabolic homeostasis. The clear role for p47phox as a driver of multiple complications that are associated with metabolic dysfunction highlights its potential significance as a therapeutic target. However, potential challenges for the successful development of targeted agents include off-target effects in normal tissues and the possible need for tissue-specific delivery systems to prevent immune suppression. Ongoing research using advanced omics approaches and sophisticated preclinical models will empower the development of the best strategies for precision targeting in different disease scenarios.

### 4.6. Cancer

p47phox plays divergent roles in cancer. It can promote tumor progression via ROS-mediated oncogenic signaling while also facilitating antitumor immunity through immune cell activation. This duality reflects its context-dependent expression and activity in tumor cells, stromal cells, and immune infiltrates. These factors make it a complex yet promising therapeutic target [108]. p47phox-driven ROS in malignant cells sustain signaling through the PI3K/AKT and MAPK pathways to drive proliferation, survival, metastasis, and chemoresistance [109]. Conversely, in immune cells, p47phox-derived ROS enhance cytotoxic T-cell and macrophage responses against tumors. Studies in *NCF1*^−^/^−^ mice suggest that p47phox deficiency modulates immune responses in cancer, potentially affecting tumor progression through altered ROS signaling [110]. These opposing effects suggest that targeted p47phox modulation could potentially enhance immunotherapy while suppressing tumor-promoting ROS.

The current evidence suggests that p47phox activity has both recurrent and distinct consequences across different malignancies. In melanoma, elevated *NCF1* expression correlates with increased basal ROS generation, enhanced BRAF/MEK/ERK signaling and elevated metastatic burden [111,112]. In estrogen receptor-positive (ER+) and triple-negative breast cancer cells, data indicate that p47phox amplifies ROS production to sustain HER2/Neu and EGFR signaling [113]. In mammary tumor models, *NCF1*^−^/^−^ may reduce tumor progression through altered NOX activity by affecting MMP-9 and VEGF [114]. p47phox also contributes to chemoresistance and studies suggest that it and other NOXs promote aggressive cancer pathophysiology through ROS-mediated signaling [114,115,116,117].

The Wnt/β-catenin signaling cascade is an important driver of epithelial-to-mesenchymal transition (EMT) and metastasis in colorectal cancer. Evidence suggests that p47phox-driven ROS regulate both of these processes [118]. Knockout of the *NCF1* gene in the *ApcMin* mouse model results in fewer intestinal adenomas and reduced basal ROS levels [110]. p47phox has also been shown to activate NF-κB in tumor-associated macrophages, which promotes chronic inflammation and tumor progression [119]. However, its role in immune system dynamics is complex. p47phox has also been shown to boost ROS in neutrophils, macrophages, and CD8+ T cells, which could enhance antitumor immunity [120].

The tumor microenvironment (TME) also plays a role in modulating p47phox activity. Hypoxia and cytokine signaling have been shown to amplify its activity in stromal fibroblasts and endothelial cells. This has been linked to angiogenesis and metastasis [121,122,123,124]. Studies conducted in preclinical models of pancreatic cancer demonstrated that p47phox increases ROS generation in stromal cells and promotes desmoplasia and chemoresistance. These findings support its potential promise as a therapeutic target [125,126]. However, the ability of p47phox modulation to yield therapeutic benefit appears to be malignancy-specific and context dependent. The known immunomodulatory effects of p47phox redox-driven signaling indicate that there may be an opportunity for synergy between p47phox stimulators and immune checkpoint inhibitors [127]. Future studies focused on defining the intricate roles of p47phox in malignant pathogenesis will enable the development of optimized therapeutic strategies.

## 5. Therapeutic Potential of Targeting p47phox

### 5.1. p47phox Inhibitors

**Figure 3:**
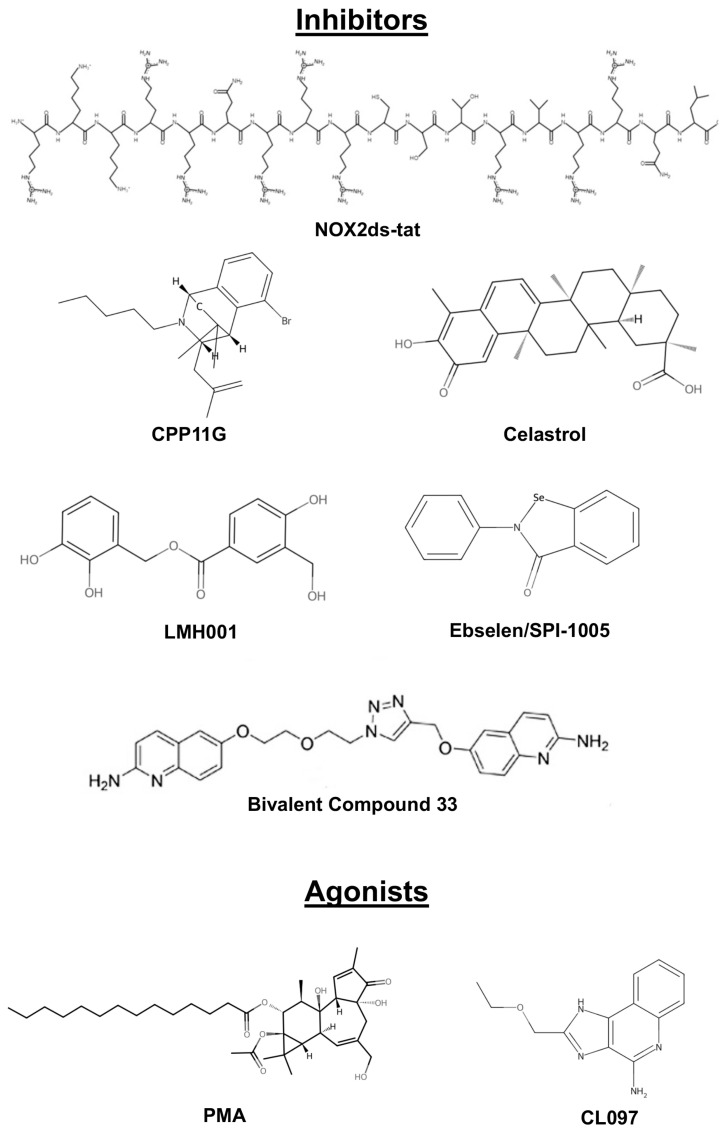
Chemical structures of representative NOX2 pathway modulators.

Inhibiting p47phox is a rational therapeutic strategy to reduce oxidative stress for diseases where excessive ROS generation drives pathology, including cardiovascular disease, RA, neurodegeneration, metabolic disorders, and cancer. To date, the development of p47phox inhibitors has largely focused on disrupting its protein–protein interactions (PPIs) within the NOX2 complex (Table 4, Figure 3). Targeting the p47phox–p22phox interface has been a major focus as this approach may reduce the probability of off-target effects on other NOX isoforms or cellular pathways [128]. Peptide-based inhibitors like NOX2ds-tat have emerged as highly specific tools that mimic the PRR of p22phox to block p47phox’s SH3 domain interactions [129,130]. In a study conducted in angiotensin II-induced hypertensive mice, NOX2ds-tat treatment reduced vascular ROS, lowered systolic blood pressure and significantly decreased the atherosclerotic plaque burden [131]. It also demonstrated therapeutic activity with respect to vascular compensation in a model of diet-induced obesity that mimicked a diabetic phenotype [132]. Similarly, NOX2ds-tat improved inflammation following traumatic brain injury, as well as pain-related behavior associated with spinal cord injuries, in preclinical studies [133,134]. Despite these encouraging results, there are challenges that may ultimately limit the successful clinical development of NOX2ds-tat and other peptide inhibitors including rapid renal clearance, limited intracellular penetration, susceptibility to proteolytic degradation, and lack of isoform specificity [129,130,135,136]. Further structure modification and innovative formulations could potentially improve its drug-like properties and make it a stronger candidate for clinical testing, but additional research is needed to determine this.

The current consensus is that small molecule inhibitors are likely to offer greater clinical feasibility over peptide-based targeting. CPP11G and CPP11H are two small molecule compounds that were designed to disrupt p47phox–p22phox binding. A preclinical study conducted with these agents in a vascular dysfunction model showed that they enhanced vascular function by lowering endothelial ROS, improving NO bioavailability, and restoring hind-limb blood flow [137]. As was learned from the preclinical evaluation of NOXA2ds-tat, there are also challenges that will likely limit the potential clinical development of CPP11G and CPP11H. These include poor solubility, potential selectivity issues, high dosing requirements and challenges inherent to PPI inhibitors. However, additional studies are required to determine if advancement into human clinical trials is warranted.

Celastrol and Ebselen are two additional early small molecule inhibitors that have demonstrated p47phox-inhibitory effects by disrupting p47phox–p22phox interactions. Celastrol is a triterpenoid compound that yields significant preclinical efficacy in multiple disease models, including AD, RA, cancer, metabolic disorders, and vascular remodeling disorders, among others [138,139,140,141,142,143]. Despite these indications of therapeutic activity, there are concerns about its long-term clinical safety. Celastrol has significant off-target effects including the broad inhibition of heat shock proteins and NF-κB. Treatment with Celastrol also resulted in liver enzyme elevations, and the potential negative effects of chronic Celastrol administration on liver function require further evaluation. Innovative delivery systems may help to improve the therapeutic selectivity and safety of Celastrol [144].

The selenium-based agent Ebselen is another example of a small molecule p47phox inhibitor. Like Celastrol, Ebselen has demonstrated preclinical therapeutic activity for a wide array of indications including cancer, diabetes, neurological disorders, hearing loss, COVID-19 and others [145,146,147,148,149]. Although treatment with Ebselen clearly decreases oxidative stress, it appears to function as a general antioxidant with limited specificity for p47phox-dependent ROS. Nonetheless, Ebselen (SPI-1005) has advanced into clinical trials for multiple therapeutic indications including bipolar disorder, treatment-resistant depression, Meniere’s disease, patients with cochlear implants, type I and II diabetes, COVID-19, and multiple forms of otoprotection (Table 5). Additionally, preclinical efforts are ongoing to develop optimized Ebselen analogs with fewer off-target effects based on recent structure–activity relationship (SAR) studies [150]. Further investigation is needed to determine whether these new Ebselen derivatives offer significant advantages over the parent compound that would support clinical investigation for the potential treatment of p47phox-linked diseases.

Novel SH3-domain binders such as C6 and C14 represent another class of agents that aim to antagonize p47phox function and have demonstrated promise in initial preclinical studies [151]. However, solubility issues may create challenges that hinder clinical evaluation. IP6 (inositol hexaphosphate) also showed potential activity as an inhibitor of p47phox based on its ability to disrupt membrane anchoring in an initial study [152]. However, its poor drug-like properties require optimization for further development. LMH001 is another small molecule inhibitor that has been reported to have efficacy in preclinical models of oxidative stress-related cardiovascular disease [153,154]. More recently, optimized bivalent inhibitors of p47phox-p22phox have been developed with sub-micromolar binding affinities and cellular activity. For example, compound 33 exhibited a Ki_50_ of 0.24 µM [155]. This impressive initial study provides strong support for further investigation.

### 5.2. p47phox Agonists

Augmenting p47phox activity to enhance NOX2-mediated ROS production represents a promising therapeutic strategy for conditions characterized by impaired immune responses, such as CGD, chronic infections, and certain cancers. By amplifying the oxidative burst in immune cells, p47phox agonists have the potential to bolster antimicrobial and tumoricidal activities [156]. This strategy could also be very effective for the selective killing of malignant cells that are under constitutive oxidative stress by pushing them over a threshold that triggers ROS-mediated apoptosis [157]. However, the systemic risks of excessive ROS production, including tissue damage and inflammation, necessitate precise control over agonist specificity, delivery, and dosing. Advances in molecular targeting and delivery systems are beginning to address these challenges and may offer a pathway toward safe and effective implementation.

PKC activators like PMA are potent inducers of p47phox phosphorylation at multiple serine residues (Ser303–Ser379) within its autoinhibitory domain [158]. This PTM disrupts intramolecular constraints, facilitates p47phox translocation to the membrane, and stimulates superoxide production in neutrophils. However, there are significant safety issues associated with systemic PMA administration including induction of pro-inflammatory IL-1β and liver toxicity [159]. To mitigate these effects, hydrogels and nanoparticle-encapsulated PMA formulations have been developed for targeted delivery [160]. These innovative delivery strategies are likely to be pivotal in harnessing PKC activators for clinical use.

Toll-like receptor ligands like the TLR7/8 agonist CL097 offer another mechanism to enhance p47phox function by stabilizing phosphorylation at Ser345 through the peptidyl-prolyl isomerase Pin1 [161]. Studies also suggest that immune stimulants may enhance ROS production in CGD neutrophils. This could potentially improve microbial clearance, but efficacy may vary [162]. This mechanism may also enhance immune responses in cancer. However, the effects of TLR agonists on p47phox/NOX2 are not specific. It is clear that other p47phox-independent mechanisms are contributing to their therapeutic activity in different contexts. Structure-based refinement paired with optimized delivery systems will empower the development of more selective agents. Synthetic analogs of the bacterial peptide fMLF provide another strategy to stimulate p47phox activity [163]. They engage formyl peptide receptors on phagocytes to improve residual NOX2 function in CGD neutrophils, increasing ROS production and microbial clearance. The ability of fMLF analogs to selectively activate phagocytes makes them versatile tools. However, their clinical translation requires further optimization to minimize cardiovascular risks.

Beyond these established agonists, novel approaches are expanding the therapeutic landscape. While no direct and specific small molecule activators of p47phox or NOX2 have been reported in the literature to date, this represents an emerging and intriguing area of investigation. Innovative, small molecule-based strategies that leverage targeted activation of p47phox to boost NOX2 activity could be used to enhance immune responses in cancer or directly kill ROS-addicted malignant cells. Other recent efforts have centered around gene therapy and RNA-based approaches. In preclinical studies, lentiviral delivery of functional *NCF1* restored ROS in CGD patient neutrophils and reduced infection rates and granuloma size [164]. This approach (PM359) recently advanced into a phase I clinical trial for patients with CGD (Table 5). It will be very interesting to see whether the impressive effects of *NCF1* gene restoration that were observed in the preclinical setting can be safely recapitulated in patients. If so, this would represent a groundbreaking development in the field of CGD management. Regardless of the specific modality, it is clear that the future of p47phox agonists lies in precision and personalization. Advances in nanoparticle-based delivery, optogenetics, and gene editing offer unprecedented control over ROS production. This will empower the development of therapies tailored to specific diseases and individual patient profiles. By navigating the delicate interplay between efficacy and safety, p47phox agonists hold the potential to transform the management of immune-deficient and neoplastic diseases.

## 6. Current Challenges and Future Directions

p47phox functions as a linchpin of NOX2-mediated ROS production. Its precision therapeutic targeting holds transformative potential for diseases characterized by redox dysregulation including CGD, cancer, diabetes and inflammatory disorders. However, its structural complexity, functional pleiotropy, and context-dependent roles present formidable challenges that demand innovative solutions. Overcoming these hurdles to unlock p47phox’s full therapeutic and diagnostic potential will require a convergence of advanced structural biology, precision medicine, and interdisciplinary collaboration.

The dynamic architecture of p47phox is governed by phosphorylation-induced conformational shifts and lipid interactions, which poses a significant barrier to drug design. Its autoinhibitory domain, SH3 domains, and PX domain orchestrate a tightly regulated activation process. This renders traditional small molecule inhibitors or agonists difficult to develop [165]. Moreover, p47phox shares structural homology with the regulatory subunits of other NOX isoforms (e.g., NOX1, NOX4). This complicates efforts to achieve isoform-specific modulation without off-target effects [166]. Recent advances in structural biology are beginning to illuminate druggable allosteric sites within p47phox’s flexible domains [28]. For instance, high-throughput screening coupled with fragment-based drug discovery has identified small molecules that disrupt p47phox’s protein–protein interactions with p22phox or Rac. However, specificity remains elusive due to overlapping binding interfaces [128]. Additionally, p47phox’s non-canonical role in actin remodeling via the Wiskott–Aldrich syndrome protein family verprolin homologous protein (WAVE) complex introduces further complexity as modulation may inadvertently affect immune cell migration or tumor metastasis [167]. These challenges underscore the need for next-generation structural tools like time-resolved crystallography and molecular dynamics simulations to map p47phox’s conformational landscape and design exquisitely selective therapeutics.

The context-dependent nature of p47phox’s function necessitates precise modulation tailored to specific disease states. Achieving this requires a granular understanding of p47phox’s phosphorylation states, expression patterns, and interactome across tissues and cell types. Emerging technologies including single-cell proteomics, spatial transcriptomics, and patient-derived organoid models are poised to address this need by mapping p47phox’s molecular signatures with unprecedented resolution [168]. These insights pave the way for precision therapeutics like proteolysis-targeting chimeras (PROTACs) to selectively degrade hyperactive p47phox in inflammatory contexts or covalent inhibitors that target reactive cysteine residues. Bispecific molecules offer another promising avenue to enhance specificity in cancer immunotherapy.

Therapeutic strategies targeting p47phox are advancing rapidly, propelled by innovations in drug delivery systems and gene editing technologies. Nanoparticle-based platforms like liposomes and hydrogels have been developed to facilitate localized delivery of p47phox modulators to minimize systemic toxicity. For instance, liposome-integrated hydrogel systems have shown promise for cancer treatment and tissue regeneration by enabling controlled release of therapeutic agents [169]. Optogenetic approaches may offer spatiotemporal precision in modulating p47phox activity. Studies have demonstrated that light-activated immune cells can enhance antitumor responses without inducing systemic oxidative damage. This could offer an approach for precise immune modulation [170].

Gene editing techniques, as exemplified by CRISPR-Cas9-mediated correction of the *NCF1* gene, present transformative avenues for restoring ROS production in CGD. Targeted repair strategies have successfully restored NOX function in patient-derived cells. This approach offers a potentially curative treatment that has recently advanced into Phase I clinical testing (PM359, Table 5) [171]. Combination therapies are also gaining traction. Evidence suggests that pairing p47phox inhibitors with immune checkpoint inhibitors may reduce tumor progression in murine models by simultaneously attenuating tumor-promoting ROS and enhancing T-cell activity [172]. Furthermore, exploration of the epigenetic regulation of the *NCF1* promoter may offer another therapeutic path. DNA hypomethylating agents that have been employed to therapeutically modulate gene expression in cancer cells could potentially be repurposed to reduce p47phox activity through epigenetic modifications in other diseases [173,174].

Structural biologists are currently leveraging cryo-EM and AI-driven molecular modeling to elucidate novel allosteric sites within p47phox and the NOX2 complex. For instance, recent cryo-EM studies have resolved portions of p47phox in active NOX complexes. This provides insights into its interactions and potential regulatory sites. Pharmacologists can utilize this information to design bispecific molecules and covalent inhibitors with enhanced specificity. Notably, the development of bivalent small molecule inhibitors targeting the p47phox–p22phox interaction has already shown promise in selectively inhibiting NOX2 activity [155].

Clinicians can use recent advancements in the understanding of p47phox pathobiology to design patient-specific trials informed by redox profiling and *NCF1* genotyping. Such stratification could enhance the efficacy of redox-based therapies by identifying patients who would benefit most from p47phox-targeted interventions. In parallel, the diagnostic and prognostic potential of p47phox is gaining attention, particularly in disorders like rheumatoid arthritis and CGD where altered expression or function of p47phox correlates with disease activity, treatment response, or risk of infection. Advancements in biomarker detection including the use of redox-related biomarkers also support personalized approaches to managing oxidative stress-related diseases. Moreover, exploration of the epigenetic regulation of p47phox should extend beyond DNA methylation to include non-coding RNAs (ncRNAs). Emerging evidence indicates that specific microRNAs can modulate *NCF1* expression, which offers a platform for precision modulation of p47phox activity.

In conclusion, p47phox has emerged as a pivotal regulator of redox biology. Its precision targeting offers significant opportunities to address diseases driven by oxidative dysregulation, including immunodeficiency, inflammation, metabolic disease, and cancer. Its structural complexity and context-dependent functions underscore the need for innovative technologies and cross-disciplinary collaboration to harness its therapeutic potential. Emerging research is also exploring p47phox as a potential diagnostic biomarker, although further validation is required. The integration of structural biology, precision therapeutics, and systems-level analysis positions p47phox as a promising target in redox medicine. Continued efforts to overcome current challenges could refine therapeutic strategies and advance the era of precision redox biology.

## Figures and Tables

**Table 1:** Regulation of p47phox functional activity by kinases and post-translational modifications.

Regulator	Specific Target(s)	Effect on NOX2 Activation	Relevance to Disease States
**Protein Kinase C ** **(PKC)**	Ser303, Ser304, Ser315, Ser320, Ser328	Phosphorylation at these sites induces conformational changes in p47phox, facilitating its interaction with p22phox and activation of NOX2	Implicated in diabetic complications and cancer progression due to enhanced ROS production
**Akt ** **(Protein Kinase B)**	Ser304, Ser328	Phosphorylation by Akt sustains NOX2 activation, prolonging ROS output	Associated with neurodegenerative diseases where prolonged ROS contributes to neuronal damage
**p38 MAPK**	Ser345	Phosphorylation at Ser345 primes p47phox for subsequent phosphorylation events, enhancing NOX2 activation	Relevant in inflammatory disorders such as rheumatoid arthritis and inflammatory bowel disease
**Ubiquitination**	Lysine residues (specific sites not fully characterized)	Ubiquitination targets p47phox for proteasomal degradation, reducing its availability for NOX2 complex assembly	Modulates severity in CGD by regulating p47phox levels
**Acetylation**	Lysine residues (specific sites not fully characterized)	Acetylation may influence p47phox stability and interaction with other proteins, potentially affecting NOX2 activity	Emerging role in metabolic stress responses; further research needed to elucidate specific mechanisms

**Table 2:** p47phox’s role in disease pathogenesis.

Disease	p47phox Role	Impact of NCF1 Deficiency	Preclinical Outcomes (NCF1^−^/^−^ Models)	Human Evidence
**Diabetic Nephropathy**	Increases glomerular ROS	Reduction in ROS	↓ Albuminuria, improved GFR	↑ NCF1 in renal biopsies ↑ 4-HNE
**Diabetic Retinopathy**	Drives vascular ROS	Reduction in ROS	↓ Vascular leakage, capillary occlusion	↑ NCF1 in retinal endothelium, ↑ nitrotyrosine
**Diabetic Neuropathy**	Promotes nerve ROS	Reduction in ROS	↑ Nerve conduction velocity, ↓ pain hypersensitivity	↑ NCF1 in peripheral nerves, ↑ lipid peroxidation
**NAFLD/Obesity**	Amplifies hepatic/adipose ROS	Reduction in hepatic ROS	↓ Lipid accumulation, ↓ fibrosis	↑ NCF1 in visceral adipose ↑ ROS markers
**Chronic ** **Granulomatous ** **Disease ** **(CGD)**	Impairs neutrophil ROS production	Reduction in ROS	↑ Susceptibility to *Aspergillus* and *S. aureus*, ↓ microbial clearance	↓ NCF1 mutations in PBMCs, ↑ infection rates, ↓ superoxide levels
**Rheumatoid ** **Arthritis** ** (RA)**	Drives synovial ROS	Reduction in ROS	↓ Synovial inflammation, ↓ cartilage erosion	↑ NCF1 in synovial biopsies, ↑ malondialdehyde, ↑ IL-6
**Inflammatory** ** Bowel ** **Disease ** **(Crohn’s Disease)**	Supports gut microbial ROS	Reduction in ROS	↑ Gut permeability, ↑ Clostridium overgrowth, ↑ colitis severity	↓ NCF1 in Crohn’s flares, ↑ IL-1β, ↓ microbial control
**Inflammatory** ** Bowel ** **Disease** ** (Ulcerative Colitis)**	Amplifies mucosal ROS	Reduction in ROS	↓ Mucosal damage, ↓ inflammatory cytokines	↑ NCF1 in severe UC, ↑ ROS markers, ↑ TNF-α
**Systemic ** **Lupus ** **Erythematosus** ** (SLE)**	Promotes autoantigen ROS	Reduction in ROS	↓ Immune complex deposition, ↓ renal damage	↑ NCF1 in lupus nephritis, ↑ 8-OHdG, ↑ anti-dsDNA antibodies
**Melanoma**	Enhances immunosuppressive ROS	Reduction in ROS	↓ Tumor growth, ↑ T-cell infiltration by	↑ NCF1 in tumor-associated macrophages, ↑ MDSC markers
**Lymphoma**	Drives tumor-promoting ROS	Reduction in ROS	↑ Tumor cell lysis, ↓ tumor burden	↑ NCF1 in lymphoma biopsies, ↑ ROS-driven immunosuppression
**Pancreatic Cancer**	Promotes tumoricidal resistance ROS	Reduction in ROS	↑ Macrophage tumoricidal activity, ↓ tumor burden	↑ NCF1 in pancreatic tumors, ↑ ROS-driven PD-L1 expression
**Breast Cancer**	Enhances tumor-promoting ROS	Reduction in ROS	↓ Tumor progression, ↑ immune cell infiltration	↑ NCF1 in breast tumor stroma, ↑ ROS-driven VEGF expression
**Colorectal Cancer**	Drives immunosuppressive ROS	Reduction in ROS	↓ Tumor growth, ↓ metastasis	↑ NCF1 in colorectal tumors, ↑ ROS-driven MDSC activity
**Psoriasis**	Promotes keratinocyte ROS	Reduction in ROS	↓ Epidermal hyperplasia; ↓ inflammatory infiltration	NCF1 mutations associated with increased susceptibility
**Asthma**	Enhances airway epithelial ROS	Reduction in ROS	Airway inflammation; ↓ eosinophil infiltration	↑ NCF1 expression in bronchial biopsies; ↑ oxidative stress markers
**Multiple Sclerosis (MS)**	Mediates CNS inflammation via ROS	Reduction in ROS	↓ Demyelination; ↓ neuroinflammation	↑ NCF1 expression in active lesions; ↑ oxidative damage markers
**Parkinson’s Disease**	Contributes to dopaminergic neuron degeneration via ROS	Reduction in ROS	↓ Neuronal loss; ↑ motor function	↑ NCF1 in substantia nigra; ↑ oxidative stress markers

**Table 3:** Disease-specific signaling pathways involving p47phox.

Disease	Signaling Pathway(s)	ROS-Mediated Effect	Downstream Consequences
**Chronic ** **Granulomatous ** **Disease** ** (CGD)**	Impaired NF-κB, IL-1β	Deficient ROS production leading to impaired microbial killing	Recurrent bacterial and fungal infections, granuloma formation, hyperinflammation
**Cardiovascular** ** Diseases**	TGF-β, NF-κB	Oxidation of LDL, promotion of atherosclerotic plaque formation	Endothelial dysfunction, vascular inflammation, plaque progression
**Neurodegenerative Diseases**	PI3K/Akt, MAPK	Enhanced microglial activation, increased α-synuclein aggregation	Dopaminergic neuronal loss, cognitive decline
**Inflammatory ** **Disorders**	NF-κB, IL-6, TNF-α	Amplification of synovial inflammation via ROS production	Joint destruction, cartilage degradation
**Metabolic ** **Disorders**	PKC, MAPK, TGF-β	Induction of glomerular oxidative stress and fibrosis	Progression of diabetic nephropathy and retinopathy
**Cancer**	PI3K/Akt, Wnt/β-catenin	Promotion of epithelial-mesenchymal transition (EMT), increased proliferation	Tumor growth, invasion, metastasis

**Table 4:** p47phox-targeted therapeutics.

Strategy	Agent/Example	Target/Mechanism	Preclinical Efficacy	Challenges
**Inhibitors** **(Peptides)**	NOX2ds-tat	Inhibit p47phox-p22phox PPI	Reduced vascular ROS and inflammation; improved hind-limb perfusion in ischemic models.	Rapid clearance, limited bioavailability
**Inhibitors ** **(Small Molecules)**	CPP11G CPP11H Celastrol Ebselen/SPI-1005 LMH001 C6 C14 Bivalent inhibitors	Disrupt p47phox-PPI Redox modulation	Decreased synovial ROS; neuroprotection in models of neurodegeneration	Off-target effects; hepatotoxicity concerns
**Agonists** ** (Gene Therapy)**	Lentiviral NCF1 PM359	Restore NCF1 expression to reconstitute NADPH oxidase activity	Reduced infection rates and granuloma formation in CGD mouse models	Potential genomic instability; variable transduction efficiency
**Agonists ** **(PKC Activators)**	Phorbol 12-myristate 13-acetate (PMA)	Induce p47phox phosphorylation to activate NADPH oxidase	Enhanced microbial clearance; increased tumor cell lysis in vitro	Systemic toxicity; pro-inflammatory responses
**Agonists ** **(TLR Ligands)**	CL097	Activate TLR7/8 pathways; modulate Pin1 and Ser345 phosphorylation of p47phox	Stimulated plasmacytoid dendritic cell activation; increased cytokine production; potential antitumor effects	Short half-life; challenges in targeted delivery

**Table 5:** Clinical trial status of NOX-targeted therapies.

Clinical Development Stage	Agent/Target	Disease Indication	Clinicaltrials.gov NCT #
**Phase II**	Sentanaxib (GKT137831) NOX1/4 inhibitor	Idiopathic pulmonary fibrosis Type 2 diabetes & albuminuria Biliary cholangitis Alport syndrome	NCT03865927 NCT02010242 NCT03226067 NCT06274489
**Phase II**	APX-115 Pan-NOX inhibitor	Type 2 diabetes with nephropathy COVID-19 Acute kidney injury	NCT04534439 NCT04880109 NCT05758896
**Phase II/III**	SPI-1005/Ebselen NOX2 inhibitor	Bipolar disorder Treatment-resistant depression Meniere’s disease Cochlear implant Type I & II diabetes COVID-19 Otoprotection	NCT03013400 NCT05117710 NCT06859788, NCT03325790 NCT06340633 NCT00762671 NCT04484025, NCT04483973 NCT01444846, NCT01451853, NCT02779192
**Phase I**	PM359 NCF1 gene therapy	Chronic granulomatous disease	NCT06559176

## Data Availability

As this is a review article, no new data were created. Data availability is not applicable.

## Abbreviations

The following abbreviations are used in this manuscript:

NOX2	NADPH oxidase 2
CGD	Chronic granulomatous disease
ROS	Reactive oxygen species
NOX	NADPH oxidase
PRR	Proline-rich region
O_2_•^−^	Superoxide radical
H_2_O_2_	Hydrogen peroxide
OH•	Hydroxyl radical
HOCl	Hypochlorous acid
NCF1	Neutrophil cytosolic factor 1
PX	Phox homology domain
SH3	Src homology domain 3
PKC	Protein kinase C
NMR	Nuclear magnetic resonance
Cryo-EM	Cryo-electron microscopy
PTM	Post-translational modification
UPS	Ubiquitin proteasome system
PAMP	Pathogen-associated molecular pattern
DAMP	Damage-associated molecular pattern
AIR	Autoinhibitory region
FRET	Fluorescence resonance energy transfer
BRET	Bioluminescence resonance energy transfer
PPI	Protein–protein interaction
TNF-α	Tumor necrosis factor alpha
PKA	Protein kinase A
CK2	Casein kinase 2
RA	Rheumatoid arthritis
AD	Alzheimer’s disease
PD	Parkinson’s disease
IBD	Inflammatory bowel disease
VEGF	Vascular endothelial growth factor
MMP	Matrix metalloproteinases
TGF-β	Transforming growth factor beta
NF-κB	Nuclear factor kappa B
NAFLD	Non-alcoholic fatty liver disease
PRR	Pattern recognition receptor
SAR	Structure–activity relationship
IP6	Inositol hexaphosphate
WAVE	Wiskott–Aldrich syndrome protein family verprolin homologous protein
PROTAC	Proteolysis targeting chimera
